# Determinants for the development of creative tourism: A stakeholder perspective

**DOI:** 10.1016/j.heliyon.2024.e33727

**Published:** 2024-06-26

**Authors:** Ting Liu, IokTeng Esther Kou

**Affiliations:** a Guangdong Polytechnic of Science and Technology, Zhuhai, China; b Faculty of International Tourism and Management, City University of Macau, Macau, China

**Keywords:** Creative tourism, Stakeholder theory, Synergies, Greater bay area, Sustainable development

## Abstract

This study aims to investigate the determinants that contribute to the sustainable development of creative tourism, a transformative shift from traditional cultural tourism which possess high economic potential. Grounded in stakeholder theory, a qualitative approach was employed to explore the perspectives of 23 existing suppliers of creative tourism in the Greater Bay Area, China, through semi-structured, in-depth interviews with key management in exiting business, this study uncovers determinants for the development of creative tourism. The findings shed light on the significance of fostering creative synergies and their implications for sustainable growth in the industry. While a positive attitude from suppliers is crucial, this study emphasizes the importance of mutual understanding of creativity to generate momentum, integration of local culture and regional knowledge, and the development of creative products. Moreover, removing barriers faced by suppliers such as a lack of financial support, insufficient government policies and regulations, and inadequate infrastructure is imperative for sustainable development. This research significantly contributes to the existing body of knowledge by providing an overview of the current research status on suppliers' perspectives towards creative tourism. It offers valuable directions for future scholarly inquiry in this field. Moreover, the research offers valuable insights that can assist policymakers and destination planners in developing efficient policies and sustainable planning approaches.

## 1 Introduction

The COVID-19 pandemic has had significant effects on the worldwide tourism sector, leading the United Nations' 2021 tourism recovery guide to highlight the importance of culture and creativity in stimulating the growth of tourism [1]. Creative tourism, also known as Cultural and Creative tourism in China, has become a significant area of focus, leading to advancements in technology and institutions within the tourism industry. It is increasingly acknowledged as a key component of economic development strategies on a global scale [[2], [3], [4]] which require additional research attention. While previous research has explored creative tourism, there is still a significant gap in the literature concerning the perspectives of businesses towards its advancement, as well as the factors and obstacles that impact it within an Asian context. To comprehensively address this gap, it is crucial to understand the perspectives and experiences of cultural and creative enterprises. By examining the perspectives, difficulties, approaches, and potential of cultural and creative applications, we can enhance our comprehension of the contribution made by these applications in stimulating the expansion of tourism and fostering economic progress.

The objective of this study is to address the existing research void by providing insights into the viewpoints and encounters of creative enterprises in the realm of tourism. Immersive and participatory experiences have propelled creative tourism into the spotlight, positioning it as a highly promising segment in the expansive realm of the tourism industry. This study focuses on the Greater Bay Area in China, a region renowned for its rich cultural heritage, diverse creative industries, and rapid economic growth. Understanding the determinants for the development of creative tourism in this regional context is crucial for sustaining and enhancing its economic potential. Through an examination of their experiences, we seek to uncover valuable insights into the challenges they face, the strategies they employ, and the opportunities they identify. This comprehensive understanding will contribute to a more holistic view of the role of cultural and creative applications in driving tourism growth and economic development. By recognizing the importance of understanding the perspectives and experiences of cultural and creative enterprises, this study goes beyond a mere analysis of external factors and aims to provide valuable insights. The findings will offer practical implications for industry stakeholders, policymakers, and researchers, aiding in the development of effective strategies, best practices, and collaborative initiatives. Ultimately, this research aims to address the following research problems to foster a sustainable creative tourism sector.

● To what extent do companies that have participated in cultural and creative activities benefit from their involvement?
● What are the attitudes of experienced managers and their involvement in cultural and creative work on the impact of these activities?
● What are the determinants for the development of creative tourism from the perspective of managing creative businesses?

## 2 Literature review

### 2.1 Creative tourism

The tourism industry has undergone a transformative shift from traditional cultural tourism to creative cultural tourism, driven by evolving market demands [5]. In response, culture and creativity have gained significant importance in promoting sustainable tourism development [6]. Over the past decade, creative tourism has emerged as a vibrant trend within China's tourism industry, giving rise to diverse forms of cultural and creative expressions encompassing products, facilities, landscapes, activities, and communities [7]. This transformation can be attributed to the widespread dissemination of innovative ideas and the implementation of relevant national policies [8].The concept of cultural tourism as a subject of academic research emerged with the rise of leisure tourism after World War II [9]. The World Tourism Organization defines cultural tourism as a visit to a cultural attraction in a city or country, outside of one's usual place of residence, with the purpose of obtaining new information and experiences that fulfill cultural needs. It also includes all activities that involve visiting specific cultural attractions. The expansion of cultural tourism is apparent in several emerging sectors, including heritage tourism, art tourism, gastronomic tourism, film tourism, and creative tourism [10].

Creative tourism was first recognized as a unique category of tourism in 1993 Pearce and Butler [11], was designed to cater to the needs of tourists seeking more engaging and participatory experiences beyond the passive nature of traditional cultural tourism. These tourists desire to explore and develop their creative potential by actively engaging with local communities [12]. Creative tourism has been hailed as a novel approach within cultural tourism that can meet the needs of these discerning travelers [13,14]. Moreover, in an increasingly competitive tourism industry, creativity is also being leveraged to enhance traditional cultural tourism experiences [15]. In contrast to traditional cultural tourism, which typically centers on fixed and observable heritage, creative tourism places emphasis on immersive encounters with everyday life, intangible cultural elements, and the acquisition of fresh insights through active participation in local communities [16].

Creative tourism provides a deeply engaging encounter that is deeply rooted in cultural heritage and delivered in a manner that is both imaginative and pioneering [17]. It distinguishes itself from traditional cultural tourism destinations and creative districts by utilizing creative vehicles to showcase and activate inherent cultural connotations [13]. The development of cultural and creative industries involves four effective approaches: borrowing and transplanting, superimposing and combining, adapting and innovating, and integrating and innovating [18]. These approaches recognize culture as the connotation, industry as the carrier, and creativity as the soul [19]. Tourism and creativity are increasingly intertwined on multiple levels, with tourism serving as a key driver of economic growth in the cultural and creative sectors [20]. In creative tourism, culture and creativity are complementary and mutually reinforcing [21].The United Kingdom, known as the birthplace of the creative industries concept, has been at the forefront of promoting creative industries through policy initiatives [22]. With 31 World Heritage Sites and numerous historic and cultural buildings, palaces, castles, mansions, and churches, the UK showcases a rich tapestry of cultural and creative assets [23,24]. In Portugal, cultural and creative industries contribute to 2.8 % of the total Gross Value Added (GVA), encompassing traditional and creatively expressed themes such as crafts, fine arts, customs and products, festivals and events, folklore, legends, novels, music, and dance [23].

Creative tourism has emerged as a novel form of cultural tourism in Thailand, where culture forms the core concept of gastronomic and cultural tourism, making creative tourism an inevitable extension [2]. In the context of China, cultural and creative industries are characterized as clusters of industries that offer cultural experiences to the general public [25]. These industries rely on creativity, creation, and innovation as fundamental mechanisms. Cultural content and creative output are considered core values, while intellectual property achievements and consumption represent transactional features. The sectors include arts and culture, media and publishing, broadcasting, film and television, software development, internet and computer services, advertising and exhibitions, art trading and design services, tourism and leisure, and other associated services [26]. Creative tourism has become a focal point of economic development in the Guangdong-Hong Kong-Macao Greater Bay Area, with the cities joining forces to promote integrated development through initiatives like the Guangdong-Hong Kong-Macao Greater Bay Area Cultural and Creative Design Competition. While research on creative tourism has explored the experiences of participating tourists, factors influencing its development, and the reasons for its slow growth, the flourishing of creative tourism requires input and perspectives from various stakeholders. The academic field recognizes the significance of multi-stakeholder inquiry through stakeholder theory [27].

### 2.2 Stakeholder theory

Stakeholder theory, introduced by Freeman and Thomlinson [27], has revolutionized the field of tourism development, transforming it into a dynamic and inclusive process. In a bustling tourist destination, various groups and individuals possess the power to influence the direction and success of tourism initiatives [28]. These stakeholders, including local residents, tourists, local governments, and tourism enterprises, play a pivotal role in shaping the creative tourism industry [29]. In fact, it is essential to give consideration to the viewpoints and concerns of the diverse range of stakeholders involved and encourage their active engagement [30], as emphasized by scholar such as Nicholas, Thapa, and others that stakeholders’ positive and negative perspective hold towards tourism development is crucial [31]. To ensure the inclusion of all stakeholders' perspectives and interests, theories based on stakeholder analysis have emerged as valuable tools [32]. For instance, the stakeholder salience model provides a comprehensive framework for identifying, categorizing, and prioritizing stakeholders based on their level of authority and immediacy [33]. The application of this model to the creative tourism industry allows for the recognition and prioritization of the interests of different stakeholders, including local residents, tourism enterprises, and the government [34].

Furthermore, stakeholder theory offers valuable insights into the roles played by various stakeholders in advancing the cause of sustainable tourism development. Exemplifying this concept are community-based tourism initiatives, which highlight the participation of local residents as stakeholders in the creation of tourism products and services. Empowering local communities in this manner not only enhances their economic well-being but also safeguards the preservation of cultural and natural heritage [35]. Similarly, considering tourists as stakeholders in sustainable tourism development encourages responsible and sustainable practices, safeguarding the destinations they visit [36].

The stakeholder theory brings attention to the intricate connections between businesses and the communities they operate in, emphasizing the need to consider a wide array of interests when making decisions. This theory acknowledges that businesses are intertwined with complex social, economic, and environmental systems, rather than existing in isolation. At its core, the stakeholder theory recognizes that the lasting success of a business depends on the well-being of these stakeholders, highlighting the importance of prioritizing sustainability over immediate financial gains. By recognizing the significance of considering the interests of all stakeholders, including local communities, natural resources, cultural heritage, and the environment, businesses can develop strategies that harmonize economic, social, and environmental aspects. This approach is fundamental to ensuring the long-term viability of the tourism sector and safeguarding the welfare of the communities and ecosystems it relies on.

### 2.3 The significance of enterprises' perspective

The role of enterprises in various industries, including tourism, has gained significant attention since the 1970s (Boğan & Sarıışık, 2020; Pereira & Gadotti dos Anjos, 2021; Wut et al., 2021). In the context of tourism development, businesses are recognized as key players in driving regional growth and prosperity (Ateljevic, 2009). Their impact on regional development and their pivotal role have been acknowledged by scholars (Korcsmáros, 2012). Understanding the importance of enterprises' perspectives is crucial for this qualitative study. The study aims to explore the factors influencing businesses' engagement with creative tourism, and to understand their motivations and challenges. By delving into their perspectives, this research seeks to provide insights that can guide regional growth and address barriers to corporate cultural and creative development.

Engaging businesses as stakeholders in the tourism planning and development process is essential for designing effective and sustainable models for tourism growth. By incorporating their perspectives, tourism planners can align the interests of businesses with the broader industry and regional goals. Collaboration and cooperation among stakeholders can pave the way for creating a tourism sector that not only thrives economically but also respects and preserves cultural heritage, protects the environment, and benefits local communities.

Furthermore, suppliers play a crucial role in achieving sustainable development and will be a focal point of this study. Their operations and practices directly impact the environment and communities, making their perspective indispensable for understanding and implementing sustainability efforts. By collaborating with suppliers on sustainable practices, tourism businesses can enhance the overall sustainability of their operations and products [37,38], contributing to the study's objectives. Recognizing the importance of enterprises' perspectives and engaging with suppliers are fundamental aspects of this qualitative study. By understanding the motivations, challenges, and aspirations of businesses, this research aims to inform policies and strategies that support their involvement in creative tourism. Additionally, incorporating the perspectives of suppliers will provide valuable insights into sustainable practices and their impact on the environment, the needs of sustainable travelers, and the economic benefits for local communities [[39], [40], [41]]. The objective of this study is to make a valuable contribution towards the advancement of a sustainable tourism sector that brings benefits to all parties involved.

## 3 Method

Owing to the exploratory nature of the study, we adopted an empirical approach to capture the experiences and subjective realities of the stakeholders [42]. The reason for adopting qualitative approach is that it allowed for an in-depth understanding of the topic and the exploration of human behavior and rationale in real-life scenarios [43]. Specifically, qualitative research was employed to gain insights into the attitudes of corporations towards the development of the creative tourism industry, considering the lack of empirical research in this area.

### 3.1 Research context

The research focused on the Guangdong-Hong Kong-Macao Greater Bay Area (GBA), encompassing a cluster of cities in the Pearl River Delta region of China, including Guangzhou, Shenzhen, and seven other prefectures, as well as the special administrative regions of Hong Kong and Macao [44]. With its vast area of approximately 56,000 km2 and a population of around 70 million, the GBA stands out for its strong economy and high GDP per capita in China [45,46]. Given the significance of tourism in the region [47], this study focused on enterprises within the GBA to explore the potential of corporate cultural and creative initiatives in the development of the creative tourism industry. The Greater Bay Area is a dynamic and interconnected region. This region boasts a unique blend of cultural traditions, historical landmarks, and modern urban landscapes. Moreover, it serves as a hub for various creative industries, including design, architecture, fashion, film, and performing arts. The diverse cultural assets and creative resources within the GBA provide a fertile ground for the development of creative tourism.

### 3.2 Sample

The study selected companies that had developed cultural and creative products or participated in creative tourism and conducted one-to-one, face-to-face interviews with their middle and senior management [48]. Purposive sampling was employed to select respondents based on their expertise in this phenomenon [49]. Middle and senior managers with more than five years management experience were chosen as participants due to their deep involvement in the external and internal operations of businesses, enabling them to provide valuable insights at both macro and peripheral levels.

During the interviews, a structured format was employed, encompassing three primary inquiries: (1) the perspectives of corporations regarding cultural and creative industries; (2) the influences affecting the advancement of creative tourism within the participating enterprises; and (3) the challenges faced by enterprises in the development of creative tourism industries. The attitudes of enterprises towards cultural and creative industries, factors affecting the development of creative tourism, and barriers to its development were assessed based on their participation in cultural and creative products. To identify companies that have developed cultural and creative products, a search was conducted on Google.hk and Baidu.com using the keyword “cultural and creative,” and companies with a high number of online posts/comments about their cultural and creative products were selected as a sample. Additional sampling was carried out on various social media platforms, followed by a snowball sampling technique [50,51]. The study participants shared specific characteristics and experiences that were relevant to the researcher's interest and contributed to a more detailed understanding of the research questions. The inclusion criteria for the respondents in the study were as follows.

(1) Companies that have cultural and creative service/ products.
(2) Respondents with more than five years of management experience in the tourism service industry and at least one year of experience in cultural and creative work.
(3) Middle and senior management of cultural and creative enterprises.

### 3.3 Data collection

Between July and September 2022, this study recruited 23 respondents, consisting of 13 males and 10 females, who were involved in cultural and creative activities and had experience in the tourism service industry and cultural and creative work. Respondents were selected using purposive sampling to ensure their expertise was relevant to the research questions [49]. Semi-structured in-depth interviews were conducted face-to-face with each respondent, with an average duration of 45 min or more, to capture their experiences and subjective realities [42], ranging from 30 min to an hour [52]. Recruitment was carried out through emails and online communication software, such as Tencent Conference and Zoom-, to ensure the inclusion of respondents of various ages and genders, aiming to achieve the “widest possible range of perspectives” [53] and enhance the study's reliability and validity [54]. The study employed an open-ended response method to collect data, and the interview process was divided into three stages: before, during, and after the interview. Prior to the interview, respondents were informed about the study's aim to examine corporate attitudes towards cultural and creative industries [55], factors influencing the development of cultural and creative industries, and barriers to corporate creative tourism. The study additionally sought to offer suggestions to the government to facilitate and encourage corporate engagement in the advancement of creative tourism. Respondents were given reassurances that the interview was strictly intended for academic purposes and would not be utilized for any other motives. Before the interview commenced, participants provided informed consent by signing a document, demonstrating their willingness to participate in the research. Moreover, they consented to have their spoken responses recorded and transcribed for subsequent analysis [56].

During the interviews, the researcher followed a semi-structured interview guide with open-ended questions to allow participants to express their thoughts and experiences freely [57]. The questions were designed to explore the participants' attitudes towards cultural and creative industries, the factors that influenced the development of creative tourism in their companies, and the obstacles they encountered in this process. The researcher actively listened to the participants, asked probing questions for clarification or elaboration, and encouraged them to provide detailed examples and anecdotes to support their responses [58]. The interviews were conducted in Mandarin and Cantonese Chinese, the primary language used in the GBA, to ensure clear communication and accurate understanding.

After each interview, the audio recordings were transcribed verbatim, and the transcripts were checked for accuracy. The researcher also made detailed field notes during and after each interview to record non-verbal cues, observations, and reflections to provide additional context to the data [51]. The transcripts and field notes were then analyzed using thematic analysis to identify patterns, themes, and categories within the data [59]. The analysis involved iterative processes of coding, categorizing, and interpreting the data to derive meaningful findings [60]. The researcher conducted the analysis manually, using a combination of inductive and deductive approaches to allow for the emergence of new themes while also considering pre-existing theoretical frameworks [61].

### 3.4 Data analysis

To analyze the collected data, a thematic analysis approach was adopted, following the guidelines outlined by Braun and Clarke [59]. The transcripts of the interviews were carefully reviewed, and significant patterns, themes, and categories were identified. The analysis involved iterative processes of coding, categorizing, and interpreting the data to derive meaningful findings [60]. The researcher conducted the analysis manually, using a combination of inductive and deductive approaches to allow for the emergence of new themes while also considering pre-existing theoretical frameworks [61]. In order to enhance the credibility and robustness of the research, various measures were implemented. To validate the accuracy and interpretation of participants' responses, member checking was carried out by sharing the initial findings with a selected group of participants [51]. Peer debriefing was carried out by discussing the research process and findings with colleagues to gain alternative perspectives and ensure the researcher's reflexivity [62]. Thick descriptions were provided in the research report to enhance the transferability of the findings to other contexts and enable readers to assess the applicability of the results [63].

The data analysis process aimed to uncover key themes and patterns in the attitudes, factors, and barriers related to corporate participation in the development of the creative tourism industry. By employing a rigorous and systematic approach, the analysis sought to provide valuable insights into the experiences and perspectives of corporations in the Guangdong-Hong Kong-Macao Greater Bay Area.

## 4 Findings

### 4.1 Determinants for cultivating creative synergies

#### 4.1.1 Positive attitudes towards development

Cultural and creative enterprises play a vital role in the development of cultural and creative industries [64]. As the main participants in cultural and creative activities, they are responsible for the production and distribution of cultural and creative products, making them a fundamental unit for the development of these industries. In the Guangdong, Hong Kong, and Macao Greater Bay Area, in addition to companies, non-profit making associations also have played a significant role in bridging the gap between cultural and creative enterprises, effectively joining forces to develop cultural and creative products. The findings of this research are consistent with previous studies that highlight the importance of corporate behavior and support for local development [65]. Yet, it is crucial to understand that respondents emphasized the importance of collaboration between cultural and creative enterprises, and associations. They also emphasized the need for innovative, locally-inspired products to promote creative tourism. One respondent stated, “The association acts as a platform for bridging the gap between cultural and creative enterprises, and we work together with them, especially in government-promoted cultural and creative projects. We aim to integrate local characteristics into cultural goods or tourism products through the creative designs of designers” (R5). This highlights the significance of collaboration between cultural and creative enterprises and the integration of local characteristics into innovative products [66]. Another respondent discussed the importance of creating platforms for young people to showcase their cultural and creative products, stating, “We should organize cultural and creative bazaars to provide young people with a platform for exchange. Through interpretation, we can turn the bazaar into a renowned carnival in Guangzhou” (R6). This highlights the potential of such events to promote creative tourism in the region. Overall, the respondents' attitudes towards the development of creative tourism underscore the importance of collaboration between cultural and creative enterprises, as well as the need for innovative and locally-inspired products to promote the growth of cultural and creative industries.

#### 4.1.2 Mutual understanding of creativity to generate momentum

The research findings reveal that respondents have different understandings of creativity in creative tourism. While scholars have provided various interpretations of creativity, there is no standardized definition [67]. Previous literature has classified creativity as a product, a person, an environment, and a process [68]. The creation of new products in different contexts and the generation of a large number of creative ideas determine the attractiveness of tourism and the development of innovative products [69]. Creativity drives the development of skills, products, and performance, and is integrated into tourism activities, enhancing cultural assets and moving away from traditional models, leading to increased tourism and consumption [70].

Respondents emphasized the potential for creativity to promote local cultural assets and attract visitors to the region. One respondent stated, “We hope to innovate something fun so that more visitors to Guangzhou can explore its traditional cultural handicrafts, not just its food and beverages” (R4). Another respondent highlighted the potential for creativity to drive economic growth and promote local culture through innovative tourism experiences and products, stating, “We aim to promote the local economy and culture through cultural innovation, travel experiences, and souvenirs” (R19). Finally, another respondent emphasized the importance of balancing the commercial aspects of creativity with the preservation and promotion of cultural heritage and assets, stating, “We should preserve culture as the core rather than solely catering to consumer demands in product design” (R11).

#### 4.1.3 Removing barriers to the development of creative tourism

During the interview process, participants described external and internal barriers to the development of cultural and creative industries in the past and present. External barriers included issues such as a lack of financial support, insufficient government policies and regulations, and inadequate infrastructure. Internal barriers included issues such as a lack of innovation, insufficient talent, and a lack of understanding of the market. The emergence of the epidemic was identified as the biggest obstacle to the development of cultural and creative enterprises. One respondent stated, “The government's policies are not enough to support the development of cultural and creative industries, especially the lack of financial support for enterprises” (R2). Another respondent mentioned, “The lack of proper infrastructure, such as exhibition halls and cultural spaces, has hindered the development of cultural and creative industries” (R17). The aforementioned external obstacles underscore the significance of governmental backing and financial allocation towards infrastructure development in order to foster the expansion of cultural and creative sectors.

Respondents emphasized the importance of innovation, talent, and market understanding as internal barriers to the development of cultural and creative industries. One respondent stated, “Innovation is crucial for the development of cultural and creative industries. Without innovation, we cannot create new and attractive products” (R9). Another respondent mentioned, “There is a shortage of talent in the cultural and creative industries, especially in areas such as design and marketing” (R13). These internal barriers highlight the need for investment in talent development and fostering a culture of innovation within cultural and creative enterprises.

The COVID-19 pandemic was identified as a significant barrier to the development of cultural and creative industries. Respondents mentioned the impact of lockdowns, travel restrictions, and reduced consumer spending on the industry. One respondent stated, “The pandemic has had a severe impact on our business. With travel restrictions and reduced tourism, our sales have plummeted” (R8). Another respondent mentioned, “The pandemic has disrupted our plans for events and exhibitions, leading to financial losses” (R15). These challenges highlight the need for resilience and adaptability within the cultural and creative industries to overcome external shocks. Importantly, when adopting new technology in service experience can enhance attractiveness [71] and using social media to entice customers can promote business visitation from online to offline environment [72], government supported finance in these specific purposes can facilitate development in the new tourism era.

## 5 Conclusion

This qualitative study has shed light on the determinants necessary for cultivating creative synergies and fostering the development of creative tourism in the Greater Bay Area, China. By contextualizing the research within the regional context, we have highlighted the unique cultural and creative value of the area. The participants emphasized the importance of positive attitudes towards development and collaboration among cultural and creative enterprises. Furthermore, the role of creativity in promoting local cultural assets and attracting visitors was recognized. To overcome barriers, governmental support and financial investment as well as internal efforts within cultural and creative enterprises is crucial. The findings of this study contribute to the body of knowledge on creative tourism by providing insights into the perspectives of industry stakeholders within the Guangdong, Hong Kong, and Macao Greater Bay Area. The implications of this research can inform policymakers, industry practitioners, and educators in their efforts to foster the growth of creative tourism.

While this research offers important insights into the growth of creative tourism, it also provides valuable information regarding the need for developing creative synergies and nurturing creative tourism. These determinants include both local and international collaboration, the definition of creative experience in terms of attractiveness and preservation, and supportive policy and government-led knowledge program.

This study provides valuable insights into the determinants necessary for cultivating creative synergies and fostering the development of creative tourism. The findings highlight the importance of positive attitudes, collaboration, and creativity in driving industry growth. The study also identifies external and internal barriers that need to be addressed to facilitate the development of cultural and creative industries. The implications of this research can inform policymakers, industry practitioners, and educators in their efforts to formulate effective policies and sustainable planning strategies that support the growth of creative tourism. While this study is limited to a single case, further research can be conducted to explore specific strategies and interventions that can overcome barriers and promote creative synergies within diverse creative tourism contexts.

### 5.1 Theoretical implications

This study explores the supportive attitudes of cultural and creative enterprises towards the development of cultural and creative industries, which are pivotal for the growth of the local industry. The findings align with previous research. [73], highlighting external epidemics and internal undercapitalization as significant hindrances to the development of the creative tourism industry. Similar findings were reported in the Grenada case study, emphasizing the impact of external factors such as limited funding and inadequate government support on the growth of creative tourism [74].

The participants in this study also stressed the importance of collaboration among cultural and creative enterprises. This aligns with the concept of creative clusters, which refers to geographic concentrations of interconnected firms and institutions working in the same industry [75]. Creative clusters promote collaboration, knowledge exchange, and innovation, which are crucial for the growth of cultural and creative industries [76,77]. The participants' emphasis on creating platforms for young people to showcase their products further supports the idea of fostering collaboration and creating opportunities for emerging talents. The role of creativity in creative tourism was another significant theme that emerged from the study. The participants recognized that creativity plays a vital role in attracting visitors and promoting local cultural assets. This is consistent with previous research that highlights the importance of creative products and experiences in enhancing the attractiveness of cultural tourism destinations [13]. The participants' emphasis on balancing commercial aspects with the preservation of cultural heritage reflects the need for sustainable development that respects and promotes local culture.

The study also identified external and internal barriers to the development of cultural and creative industries. External barriers, such as financial constraints and inadequate government support, highlight the importance of policy interventions and investment in infrastructure to support the growth of the industry [18]. Internal barriers, such as a lack of innovation and market understanding, point to the need for capacity-building initiatives and educational programs to develop the necessary skills within cultural and creative enterprises.

### 5.2 Practical implications

#### 5.2.1 Local and international collaboration

The findings of this qualitative study provide valuable insights into the determinants necessary for cultivating creative synergies and fostering the development of creative tourism. The participants emphasized the significance of positive attitudes towards development within cultural and creative enterprises, highlighting their fundamental role in industry growth. These enterprises play a crucial part in the production and distribution of cultural and creative products, making collaboration among them vital for promoting creative tourism. These findings align with previous studies that emphasize the importance of corporate behavior and local development support [65]. The participants also expressed the need for innovative, locally-inspired products that integrate different local characteristics into cultural goods or tourism experiences. This highlights the importance of collaboration and the incorporation of distinct regional elements to foster innovation.

Moreover, the respondents highlighted the importance of creating platforms for young people to showcase their cultural and creative products. By organizing events such as cultural and creative bazaars, the region can facilitate knowledge exchange and transform such events into renowned carnivals, thereby promoting creative tourism. These findings underscore the potential of collaborative platforms and events to attract visitors and stimulate the growth of cultural and creative industries.

#### 5.2.2 Defining creative experience in the perspective of attractiveness and preservation

The participants in this study exhibited diverse understandings of creativity in the context of creative tourism. While scholars have provided various interpretations of creativity, a standardized definition remains elusive [67]. Nonetheless, creativity plays a significant role in driving creative tourism. The creation of new products and the generation of creative ideas determine the attractiveness of tourism destinations and the development of innovative experiences [69]. The participants recognized the potential of creativity to promote local cultural assets and attract visitors to the region. A key aspect emphasized by the participants was the balance between the commercial aspects of creativity and the preservation and promotion of cultural heritage and assets. They stressed the need to prioritize the preservation of cultural elements and design products that cater not only to consumer demands but also to the authenticity of the local culture. This aligns with the idea that culture should be preserved as the core, rather than solely designing products for commercial purposes. Consequently, a nuanced approach to creativity that considers both market demands and cultural heritage is essential for sustainable creative tourism development.

#### 5.2.3 Supportive policy and government-led knowledge program

During the interviews, participants identified external and internal barriers that have hindered the development of cultural and creative industries. External barriers encompassed issues such as inadequate financial support, insufficient government policies and regulations, and a lack of necessary infrastructure. These barriers highlight the importance of government support and investment in cultural and creative industries to facilitate their growth. Governments could provide financial assistance, develop supportive policies, and invest in the necessary infrastructure to foster the development of creative tourism.

Internal barriers, on the other hand, were identified as challenges within cultural and creative enterprises themselves. These obstacles included a lack of innovation, limited understanding of the market and consumer needs, and a shortage of talent with the necessary skills. To address these internal barriers, cultural and creative enterprises should focus on nurturing innovation, understanding market dynamics, and developing creative talents. Additionally, participants emphasized the importance of educational and training programs that provide the necessary skills and knowledge to contribute effectively to the industry.

#### 5.2.4 Collaborative sustainable development strategies

While it is of utmost importance to foster collaborative endeavors among various stakeholders, it is suggested to adopt a collaborative approach to develop sustainable strategies for unparalleled creative tourism experiences, thereby ensuring sustained growth and competitiveness. This shared understanding cultivates a favorable environment for the development of creative products and services that cater to the preferences of both domestic and international tourists. Another crucial factor for the sustainable development of creative tourism lies in the integration of local culture and regional knowledge. By capitalizing on the unique cultural heritage and offering authentic experiences, destinations within the Greater Bay Area have the opportunity to distinguish themselves and allure visitors seeking immersive encounters with local traditions and contemporary creative expressions. This integration not only enriches the visitor experience but also contributes to the preservation and promotion of cultural assets. While there are certain obstacles to the development of creative tourism in the region, it is imperative for policymakers and destination planners to address these challenges and collaborate with stakeholders to establish an enabling environment for sustainable development. This may entail providing financial incentives, formulating supportive policies, and investing in infrastructure development to enhance accessibility and connectivity within the Greater Bay Area.

### 5.3 Limitation and future research

This study has certain limitations that should be acknowledged. By addressing these limitations, future research can build upon the findings and contribute to a more comprehensive understanding of the determinants for the development of creative tourism. Firstly, even Greater Bay Area is an important region for economic development in Mainland China and valuable insight has been uncovered, this study presents results of a single case which limit its generalization, and generalizing the findings to other cases should be done cautiously. Future research could explore additional cases in different regions to enable meaningful comparisons and enhance the generalizability of the conclusions. Secondly, the research on corporate creative tourism is at a preliminary stage. As a result, the conclusions drawn from this study may be limited in their scope. Future studies can delve deeper into specific aspects of corporate creative tourism to provide a more nuanced understanding and capture a broader range of perspectives. Thirdly, the survey is only based on the case of the Greater Bay Area. While this approach captured the key points, additional research in the future could expand the geographical scope and include a more diverse range of locations. By examining different regions, researchers can examine the internal and external obstacles, and outline whether the barriers and obstacles identified in the local ecosystem are unique or consistent across various contexts. Finally, the findings of the research are constrained by the sample size of 23 businesses that were examined. In future research, it would be beneficial to aggregate larger sets of diverse data to facilitate quantitative analysis from a broader perspective. By expanding the sample size, researchers can strengthen the statistical validity of the findings and gain a more comprehensive understanding of the determinants for creative tourism development. Finally, conducting more comprehensive and systematic analysis by surveying a larger sample of enterprises engaged in the development of cultural and creative industries would yield valuable insights. By including a broader range of enterprises, future studies can provide a more holistic view of the challenges and opportunities faced by stakeholders in the creative tourism industry.

## Data availability

The data associated with your study has not been deposited into any publicly available repository. It will however be made available upon request.

## CRediT authorship contribution statement

**Ting Liu:** Writing – original draft, Project administration, Formal analysis, Data curation, Resources. **IokTeng Esther Kou:** Writing – review & editing, Writing – original draft, Supervision, Formal analysis, Data curation, Conceptualization, Methodology.

## Declaration of competing interest

The authors declare that they have no known competing financial interests or personal relationships that could have appeared to influence the work reported in this paper.
